# Is Continuous Wound Infiltration a Better Option for Postoperative Pain Management after Open Nephrectomy Compared to Thoracic Epidural Analgesia?

**DOI:** 10.3390/jcm12082974

**Published:** 2023-04-19

**Authors:** François Crettenand, Nady Assayed-Leonardi, Felix Rohrer, Silvia Martinez Carrique, Beat Roth

**Affiliations:** 1 Department of Urology, Lausanne University Hospital and University of Lausanne, Rue du Bugnon 46, 1011 Lausanne, Switzerland; 2 Department of Anesthesiology, Lausanne University Hospital and University of Lausanne, 1011 Lausanne, Switzerland

**Keywords:** ERAS^®^, nephrectomy, continuous wound infiltration, kidney cancer

## Abstract

Background: Despite increasingly advanced minimally invasive percutaneous ablation techniques, surgery remains the only evidence-based therapy in curative intent for larger (>3–4 cm) renal tumors. Although minimally invasive surgery using (robotic-assisted) laparoscopic or retroperitoneoscopic approaches has gained popularity, open nephrectomy (ON) is still performed in 25% of cases, especially in tumors with central localization (partial ON) or large tumors with/without cava thrombus (total ON). As postoperative pain is one of the drawbacks of ON, our study aims to assess recovery and post-operative pain management using continuous wound infiltration (CWI) compared to thoracic epidural analgesia (TEA). Methods: Since 2012, all patients undergoing ON at our tertiary cancer center at CHUV have been included in our prospective ERAS^®^ (enhanced recovery after surgery) registry that is centrally stored in ERAS^®^ Interactive Audit System (EIAS) secured server. This study represents an analysis of all patients operated on with partial or total ON at our center between 2012 and 2022. An additional analysis was performed for the estimations of the total cost of CWI and TEA, based on the diagnosis-related group method. Results: 92 patients were included and analyzed in this analysis (n = 64 (70%) with CWI; n = 28 (30%) with TEA). Adequate oral pain control was earlier achieved in the CWI group compared to the TEA group (median 3 vs. 4 days; *p* = 0.001), whereas immediate postoperative pain relief was better in the TEA group (*p* = 0.002). Consequently, opioid use was higher in the CWI group (*p* = 0.004). Still, reported nausea was lower in the CWI group (*p* = 0.002). Median time to bowel recovery was similar in both groups (*p* = 0.03). A shorter LOS (0.5 days) was observed in patients managed with CWI, although this was not statistically significant (*p* = 0.06). The use of CWI has reduced total hospital costs by nearly 40%. Conclusions: TEA has better results in terms of postoperative pain management compared to CWI following ON. However, CWI is better tolerated, and causes less nausea and earlier recovery, which leads to a shorter length of stay. Given its simplicity and cost-effectiveness, CWI should be encouraged for ON.

## 1. Introduction

In Switzerland, the age-standardized rate for renal cell carcinoma (RCC) is 6.5 per 100,000 inhabitants, with a cumulative risk of 0.74. Worldwide, RCC represents the sixth and tenth most frequently diagnosed cancer in men and women, respectively [1]. Since its description by Robson in 1963 [2], surgery remains the treatment of choice to date and the only evidence-based therapy in terms of curative intent for RCC, especially those of larger sizes (>3–4 cm) [3]. Minimally invasive approaches like laparoscopic nephrectomy have been linked to reduced length of stay (LOS) and postoperative analgesic requirement [4,5]. However, there is still no randomized controlled trial (RCT) assessing the oncological outcomes of such a technique.

Despite a rising trend toward minimally invasive technics, open nephrectomy (ON) is still performed in a quarter of cases [6], especially in tumors with central localization for partial ON (as minimally invasive radical nephrectomy should not be performed in patients with T1 tumors for whom a partial nephrectomy is feasible by any approach [3]) or large tumors with/without cava thrombus (total ON). A major drawback of ON is postoperative pain and its known association with impaired recovery and higher postoperative complication rates [7,8]. Thoracic epidural analgesia (TEA) is a well-recognized and relatively safe postoperative pain management technic in open surgery [9]. However, its potentially serious complications, such as neuraxial abscess and hematoma or permanent neurological damages, would make pain management alternatives very attractive [10]. Moreover, TEA can cause hypotension, dizziness and fatigue that may require aminergic support and prolonged surveillance in intermediate care [11]. This results in delayed postoperative mobilization, a cornerstone of the enhanced recovery after surgery (ERAS^®^) program [12]. The present study aims to assess the impact of continuous wound infiltration (CWI) as a pain management alternative compared to TEA in terms of patient recovery following ON (partial or total) for RCC in an ERAS^®^ setting. 

## 2. Materials and Methods

### 2.1. Patient and Data Selection

All adult patients with open radical or partial nephrectomy attending our tertiary referral center between November 2012 and June 2022 were included in this cohort study. As partial and total nephrectomies were usually performed by robotic-assisted laparoscopic surgery at our hospital, only the following patients were candidates for open surgery: (a) patients in whom partial nephrectomy was not feasible by robotic-assisted laparoscopy due to anatomical conditions (e.g., centrally localized/endophytic tumors) but by open retroperitoneal technique; (b) patients with contraindication for a pneumoperitoneum (e.g., pulmonary compliance, cardiovascular issues, intracranial disease); (c) patients with extensive intra-abdominal adhesions after previous surgery; (d) patients with tumor thrombus in the vena cava. To have the best cancer treatment, all patients were discussed at our weekly tumor board together with dedicated radiologists. Whenever feasible, a robotic-assisted approach was chosen. 

Data were obtained through a prospectively maintained and centralized database archived in Amazon Web Services, Inc (AWS) secured servers located in Frankfurt am Main, Germany, and accessed through the ERAS^®^ interactive audit system (EIAS). All surgical procedures were performed within an ERAS^®^ pathway. Demographic, surgical, recovery and ERAS^®^ related data were prospectively recorded by a dedicated study nurse. Data integrity and consistency was cross-checked with a regular internal audit by a dedicated ERAS^®^ team. Age, gender, body mass index (BMI), smoking habits, oncological and recovery data, such as days in the intensive care unit (ICU), time to first bowel movement, time to mobilization, LOS, were further recorded. Postoperative complications were classified using the Clavien–Dindo classification [13]. Postoperative pain was assessed using the Visual Analogue Scale (VAS), ranging from 0 (no pain) to 10 (worst pain ever) [14]. Nausea was assessed using a similar scale ranging from 0 (no nausea) to 10 (nausea and vomiting). The administration of strong opioid “salvage therapy” either per os (Oxycodone), subcutaneously or intravenously (Morphine) was consigned for each postoperative day (POD). The detailed cost for each patient and hospital stay were retrieved within the hospital accounting database and calculated with a diagnosis-related group method (DRG) modified for Switzerland. Total operative time was defined as the time from the first sedative medication until patient extubation and transfer to the recovery room. Induction time was defined as the time from the first anesthesiology procedure required to perform the surgical intervention (preoxygenation and monitoring devices placement) until patient positioning and disinfection. Patients were allocated to two groups according to the type of postoperative pain management device: TEA vs. CWI.

### 2.2. ERAS^®^ Protocol

Since no specific ERAS^®^ guidelines were available for patients undergoing renal surgery by lumbotomy, their management was adapted from previously published guidelines for radical cystectomy [12,15]. In brief, all patients scheduled for renal surgery received preoperative counseling by a dedicated nurse, including smoking and drinking cessation as well as nutritional status evaluation. Physical activity prior to surgery was strongly advised. Patients were screened for preoperative anemia, which was corrected whenever feasible and in line with the patient blood management (PBM) policy of our institution. The concepts used are based on the previously published three-pillar matrix, which can be summarized as early anemia treatment, blood loss reduction and rational and guideline-appropriate use of transfusion [16]. Preoperative sedative medication was avoided. The patient received a carbohydrate-rich drink 2 h before surgery. Antibioprophylaxis with a second-generation cephalosporin was administered 30–60 min prior to skin incision. In the postoperative period, early mobilization (sitting upright and transferring from bed to chair) was encouraged at POD 0. From POD1 onwards, patients were encouraged to sit in a chair for at least 6 h and to walk a minimal distance of three times 150 m. Likewise, early oral feeding was promoted with freely available tea or water accompanied by broth and toast as early as 4 h after surgery, followed by a low-fiber diet on POD 1 and POD 2. Normal occidental diet resumed from POD 3. Gut motility was stimulated with chewing gum. Surgical site drainage was, whenever possible, avoided or removed early (POD 2). A urinary catheter was removed early (at the latest after 48 to 72 h postoperatively). For study purposes, adequate per os pain control was definite as a VAS ≤ 4. 

### 2.3. Anesthesiology Protocol

Every patient was evaluated in a pre-anesthetic consultation, where perioperative risks were assessed, the anesthetic strategy was established and informed consent was obtained. A standard anesthetic protocol was used according to recommendations from the ERAS^®^ Society consensus statement paper updated and published in 2015 [17]. Standard monitoring and equipment included a pulse oximeter, non-invasive blood pressure, 5-derivation ECG, Bispectral Index Saturation, neuromuscular monitoring and two peripheral venous access. Intraoperative anesthesia and analgesia were achieved by Sevoflurane or Disoprivan and fentanyl. Perioperative fluid management aimed at normovolemia, using a continuous infusion of ringer-lactate 3–5 mL/kg/h, adapted according to clinical evaluation. Nausea prophylaxis was achieved using Dexamethasone 4 mg and Droperidol 1 mg after induction and ondansetron 4 mg at the end of the intervention. Postoperative pain management was chosen at the discretion of the anesthesiologist as follows:

#### 2.3.1. TEA Placement 

TEA placement was done preoperatively at the lower thoracic level (Th. 7–10) routinely by a resident under senior supervision. Correct positioning was confirmed by a test dose of 3 mL lidocaine 1% with epinephrine 10 mcg/mL. Intraoperative pain control was achieved using bupivacaine 0.5%. Post-operatively, a continuous epidural infusion of a solution containing low-dose bupivacaine 0.0625%, Fentanyl 2 mcg/mL and Epinephrine 2 mcg/mL with additional patient-controlled boluses was used. Paracetamol (1 g every 6 h) and Metamizole (500 mg every 6 h) were concomitantly administered, with a first dose given at the end of surgery. A trained analgesia nurse daily evaluated the patient and adapted dosing and co-medication if necessary. The epidural catheter was removed on postoperative days 3 to 5, according to pain control and bowel function and after an evaluation of comfort and pain reported by the patient.

#### 2.3.2. CWI Placement 

After the closure of the deep muscle layers (transversus abdominis and obliquus internus) using No. 2 interrupted polyglycolic acid sutures, two multiperforated 12.5 cm or 25 cm incisional catheters (ON-Q^®^ Soaker Catheter^TM^, B. Braun Medical, Sempach, Switzerland) were inserted under digital control. Only thereafter, the superficial layer was subsequently closed (obliquus externus abdominis). No subcutaneous sutures were applied, and the wound was closed with a 35-wide skin stapler. A bolus dose of 2 × 10 mL of 0.2% Ropivacain (Sintetica SA, Mendrisio, Switzerland) was injected through incisional catheters. An elastomeric pump (ON-Q^®^ PainBuster, B. Braun Medical, Sempach, Switzerland) filled with 290 mL of the same anesthetic solution was then connected to the system. Ropivacain delivery rate was calibrated as 4 mL per hour for 72 h, for a total dosage of 620 mg over 3 days. CWI has not refiled once empty, therefore, its removal after this period. Paracetamol (1 g every 6 h) and Metamizole (500 mg every 6 h) were concomitantly administered. Morphine hydrochloride 0.1 mg per kilogram of body weight was available as “salvage” pain medication. 

### 2.4. Statistical Analysis

All data were anonymized prior to statistical analysis. Categorical variables were presented as numbers and proportions, and continuous variables by median and interquartile range (IQR) or mean and standard deviation (SD) as appropriate. Group differences in categorical variables and continuous variables were analyzed with a chi-square test and Kruskall–Wallis test, respectively. After testing for normality with the Kolmogorov-Smirnov test, pain and nausea scores were analyzed with one-way ANOVA or the Mann–Whitney U-test as appropriate. Statistical testing was two-sided and a *p*-value < 0.05 was considered statistically significant. Analyses were all conducted with STATA 17 (College Station, TX, USA). 

## 3. Results

**Figure 1:**
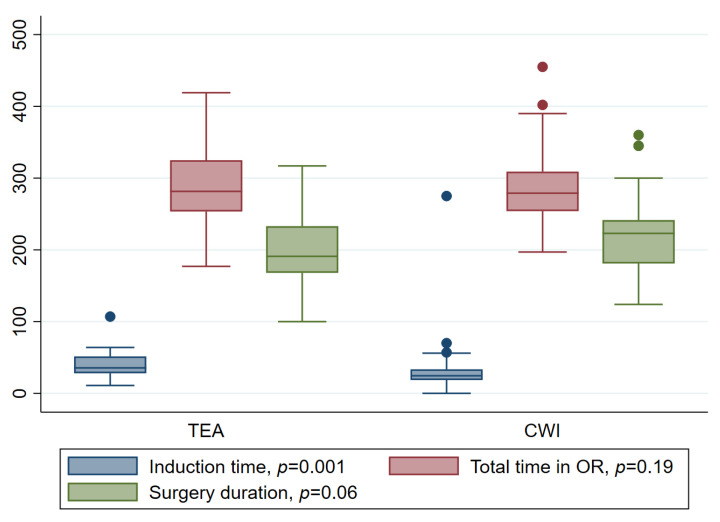
Visual comparison in surgical procedure between patients managed with thoracic epidural analgesia (TEA) and continuous wound infiltration (CWI) expressed in minutes. Total time in the operating room (OR) is the patient’s entire stay in OR. Induction time starts with the first administration of anesthesia drugs. Surgery duration is calculated from skin incision till skin closure.

A total of 94 patients were included. Two patients (2%) were excluded from the final analysis; one due to patient refusal after initial inclusion and another due to a technical problem with the epidural catheter. Thus, 64 (70%) were managed with CWI and 28 (30%) with TEA. The two groups were comparable in terms of demographics and oncological and surgical data (Table 1). Complications rates were also comparable between the groups. No complication was assessed as being related to the analgesia technique. When evaluating postoperative recovery, patients in the CWI group had their indwelling catheter removed one day earlier (median 3 days, IQR 2–4) compared to patients with TEA (median 4 days, IQR 2–5; *p* = 0.05). Adequate per os pain control was achieved within a median of 3 days (IQR 1–4) in the CWI group compared to 4 days (IQR 4–6; *p* = 0.001) in TEA group. The median time to bowel recovery was 2.5 days [IQR 2–3] in the CWI compared to 3.5 days [IQR 2–3] in the TEA group (*p* = 0.03). Patients in the CWI group were able to reach mobilization goals as described above one day earlier than those in the TEA group; this difference did, however, not reach a level of significance (*p* = 0.07). A trend toward shorter median LOS was observed in the CWI group (6 days [IQR 5–8] versus 6.5 days [IQR 5.5–9], *p* = 0.06). There was a statistically significant difference in pain score at POD 0 between the two groups (*p* = 0.002) with a mean value of 2.8 (SD 3.4) in the TEA group compared to 5.1 (SD 2.9) in the CWI group. However, as Table 2 shows, this analgesic effect tends to decrease during the days following surgery in both groups; still, a significantly better pain control with TEA persisted at POD 3 (*p* = 0.0025; Table 2). As a consequence, opioid use was significantly higher in the CWI group, in which more than half of the patients received concomitant opioid treatment at some point in the postoperative period (Table 3). Interestingly, mean self-reported nausea score was significantly lower in the CWI group during the postoperative course (Table 4). Only one patient in each group required ICU care post-operatively. In terms of cost-effectiveness, the average mean cost per patient for the total hospital stay was CHF 11200 (equivalent to USD 11800) cheaper in the CWI group than in the TEA group. This amount includes operation and operating room costs, costs for all consumable material, as well as costs for hospital stay and postoperative care until discharge. When considering time-effectiveness, the median total time in the operating room was similar in both groups (279 min. [IQR 254–309] in the CWI group vs. 282 min. [IQR 256–325] in the TEA group; *p* = 0.19). However, the mean induction time was 12 min shorter in the CWI than in the TEA group (24 min. [IQR 18–33] vs. 36 min. [IQR 28–52]; *p* = 0.001). As illustrated in Figure 1, median surgery time *per se* (including CWI catheter placement) did not significantly differ between the two groups (CWI: 233 min [IQR 180–242]; TEA: 195 min [IQR 168–233]; *p* = 0.06). 

## 4. Discussion 

Postoperative pain control remains a major challenge when managing patients undergoing ON, especially with a retroperitoneal approach. Inadequate postoperative pain control could potentially lead to impaired recovery and chronic pain [18]. While slightly better analgesia was achieved using TEA compared to CWI in our study with less use of “salvage” opioids, this did neither translate into a better postoperative nausea score nor a better bowel function recovery (Table 4). These conflicting results might be explained by the possible alterations on blood pressure leading to orthostatic hypotension in the postoperative period induced by epidural analgesia. Orthostatic hypotension, in turn, may lead to dizziness or nausea, thus reducing early mobilization after surgery, especially among patient with a mean blood pressure of less than 70 mmHg [19]. However, we could not support this hypothesis in our studied population since the range in blood pressure variation was not recorded in our dataset. Additionally, fentanyl present in the epidural perfusion may also cause nausea [20].

Forastiere et al., in a placebo-controlled, randomized controlled trial of 168 patients undergoing ON for renal cancer, showed a shorter bowel recovery time and LOS (2.1 days versus 3.2 days, *p* < 0.001) with a CWI filled with Ropivacaine solution compared to CWI with normal saline and IV morphine dispensed through a patient-controlled analgesic device. As expected, better pain control was obtained in the CWI group than in the placebo group in their study [21]. More recently, a study conducted by Gebhardt and colleagues [22] on patients scheduled for open thoracotomy and managed with either CWI or TEA were accessed for postoperative pain and postoperative recovery. The authors reported a similar pain score at POD 3 between the two groups, with a reduction of mean LOS of one day in the CWI group. Our results are consistent with the available literature in favor of CWI compared to TEA for postoperative pain management. Moreover and in line with the results of our study, a systematic review comprising 44 randomized controlled trials reported a mean reduction of the postoperative nausea and vomiting score of 45% when CWI was used instead of TEA for postoperative analgesia in major surgeries [23]. Despite better pain control with TEA, the patients felt well enough to achieve mobilization objectives and were discharged earlier. These results are consistent throughout the literature in various surgical procedures, although no comparison for open nephrectomies exists. Especially in liver surgery, no inferiority of CWI compared to TEA could be found in three RCTs [24,25,26]. Moreover, a RCT including 40 patients undergoing radical cystectomy compared analgesia and inflammatory response in patients with preperitoneal CWI vs. epidural analgesia [27]. Their results suggest that the inflammatory response was lower in the CWI group, with comparable analgesia in both groups. Even if pain control for a lumbotomy may be different than the one of a midline laparotomy approach as performed for cystectomies, the results are encouraging. CWI was not inferior in terms of pain management in our study, but reduced LOS, was much easier to handle, and was much more cost-effective. Nevertheless, the use of CWI versus TEA for lumbotomy should also be addressed in a RCT before a definitive conclusion can be drawn. The increases in opioid consumption in the CWI group should be further addressed with other components of multimodal analgesia techniques, such as the use of intraoperative Dexmedetomidine infusions, for example [28].

In the last two decades, the incidence of renal cancer increased gradually to approximately 2% a year. This effect seems to be mainly linked to a larger use of computed tomography in clinical practice leading to incidental findings. As a result, renal tumors are detected and treated at an earlier stage (lower tumor size) than 20–30 years ago [29], and partial nephrectomy (PN) is usually recommended as standard treatment for these small and localized (T1) RCC irrespective of surgical approach [3]. 

Various studies have already shown the advantages of nephron-sparing surgery in terms of oncological and non-oncological outcomes [30,31]. Anastasiadis et al. highlighted the contemporary place of ON in potentially difficult surgeries (entophytic or voluminous tumor), in case of redo surgery, or with multiple and recurrent renal tumors (hereditary renal cancer) [32]. In their 2012 national audit on PN in the United Kingdom, Fernando and colleagues showed the preponderant place of partial ON for large renal masses (>4.5 cm) and for stringent indication to nephron-sparing surgeries (single kidney, chronic kidney diseases and bilateral tumors). These results seem to be explained by longer ischemic time or more complex anatomical reconstruction with minimally invasive techniques compared to the open technique [33]. For all these reasons, ON has still a place in the management of a patient with renal cancer. In this regard, advances in open techniques are imperatively needed and remain essential. As such, CWI is cost-effective with a significant reduction of total hospital costs per case of around 40%, mainly caused by reduced OR time, reduced LOS and reduced nursing workload for surveillance. Our results represent the real-world costs of a whole hospital stay based on a national based cost allocation program and not a simple estimation of the different procedures (real costs per patient charged to the health insurance for the hospital stay). The cost reduction of CWI for open renal surgery presented here are consistent with those observed in literature for thoracic, and gynecologic surgeries using CWI [21,22,34].

The authors are aware that the present study is not without several limitations. One of the main limitations is the relatively small number of patients in this cohort study. Nevertheless, the preponderance of minimally invasive kidney surgery renders difficult to design and carry out large prospective randomized trials specifically addressing this question. Another limitation is that pain assessment is only recorded once a day in EIAS database, although performed several times per day. Thus, known daily variability in pain experienced by patients could not be considered in this analysis [35]. Unfortunately, intraoperative opioid consumption and time to first opioid dose so range in blood pressure variation are not available in our ERAS^®^ database. However, this study focuses on patient recovery like time to mobilization and first bowel movement; thus, these additional information shouldn’t have changed results significantly.

However, this study shows the numerous advantages of CWI placement compared to TEA in terms of postoperative recovery and costs. These positive effects significantly outweighed the modest negative impact of increased opioid use during the first three PODs. An important point is the fact that CWI catheter placement is simple, straightforward and can be performed and controlled by the surgeon themself, i.e., independent of anesthesia valences (as detailed in Figure 1). For all of these reasons, TEA is no longer routinely performed for open renal surgery at our tertiary center.

## 5. Conclusions

Thoracic epidural analgesia showed better results regarding post-operative pain management than continuous wound infiltration following open nephrectomy. However, continuous wound infiltration with an elastomeric pump is better tolerated with fewer nausea, earlier recovery and shorter length of hospital stay. Given its simplicity and distinct cost efficacy, its use should be encouraged when applying principles of enhanced recovery after surgery. However, randomized controlled trials should be utilized to confirm these results.

## Figures and Tables

**Table 1:** Patients’ characteristics of the two studied populations either with continuous wound infiltration (CWI) or with thoracic epidural analgesia (TEA).

	CWI	TEA	
**N**	64	28	***p***
Age—median (IQR)	62 (51.5–67.5)	63.5 (54.5–75)	0.35
Gender—n (%)			0.65
Female	22 (34.4)	11 (39.3)	
Male	42 (65.6)	17(60.7)	
Smoking—n (%)	33 (55)	19 (67)	0.25
BMI—median (IQR)	26.4 (22.9–31.1)	25.5 (22.3–30.2)	0.11
Diabetes—n (%)	11 (17.2)	6 (21.4)	0.72
Hypertension—n (%)	11 (17.7)	5 (18.2)	0.49
Procedure—n (%)			0.39
radical nephrectomy	29 (45.3)	10 (35.7)	
partial nephrectomy	35 (54.7)	18 (64.3)	
pT stages—n (%)			0.28
pT1	32 (50)	12 (42.9)	
pT2	6 (9.4)	0	
pT3	18 (28.1)	7 (25)	
pT4	1 (1.6)	0	
non-oncological	7 (10.9)	9 (32.1)	
pN stage—n (%)			0.08
pN0	17 (26.5)	8 (28.6)	
pN1	0	2 (7.2)	
pNx	40 (62.5)	9 (32.1)	
non-oncological	7 (11)	9 (32.1)	
Complications—n (%)			0.17
Clavien I	8 (12.5)	6 (21.4)	
Clavien II	13 (20.3)	7 (25)	
Clavien IIIa	7 (10.9)	0	
Clavien IIIb	1 (1.6)	2 (7.2)	
No complication	35 (54.7)	13 (46.4)	

**Table 2:** Analysis of variance (one-way ANOVA) and mean visual analogue scale (VAS) for pain score reported by CWI and TEA patients on each postoperative day (POD).

VAS Score at	Analysis of Variance		Mean VAS—(SD)	
	F	*p*	CWI	TEA
POD 0	(1, 87) = [9.95]	0.002	5.1 (2.9)	2.8 (3.4)
POD 1	(1, 88) = [5.02]	0.03	4.9 (2.6)	3.5 (2.7)
POD 2	(1, 89) = [1.48]	0.96	3.5 (2.6)	3.6 (2.8)
POD 3	(1, 87) = [4.51]	0.025	3.8 (2.5)	2.6 (2.3)

**Table 3:** Ratio of patients received a strong opioid (either per os, subcutaneously or intravenously) in days following surgery reported for two groups compared with χ^2^ test.

	CWI	TEA	
**N**	64	28	***p***
Opioids at—n (%)			
POD 0	41 (64)	0	<0.001
POD 1	41 (64)	2 (7.1)	0.004
POD 2	33 (51.5)	2 (7.1)	0.04
POD 3	27 (42.2)	5 (17.9)	0.68

**Table 4:** Impact of postoperative pain management technic on patients’ self-reported nausea (ranging from 0 to 10) on different postoperative days (PODs).

Nausea Score at			Mean Nausea Score—(SD)	
	z	*p*	CWI	TEA
POD 0	0.795	0.42	0.6 (2.1)	0.9 (2.4)
POD 1	2.730	0.02	0.8 (2.4)	2.7 (4.1)
POD 2	3.081	0.002	0.9 (2.5)	3.3 (4.4)
POD 3	3.000	0.003	0.6 (1.8)	2.1 (3.5)

## Data Availability

The data presented in this study are available on request from the corresponding author. The data are not publicly available due to ethical consideration.
